# Inhibition of Proliferation and Epithelial Mesenchymal Transition in Retinal Pigment Epithelial Cells by Heavy Chain-Hyaluronan/Pentraxin 3

**DOI:** 10.1038/srep43736

**Published:** 2017-03-02

**Authors:** Hua He, Ajay E. Kuriyan, Chen-Wei Su, Megha Mahabole, Yuan Zhang, Ying-Ting Zhu, Harry W. Flynn, Jean-Marie Parel, Scheffer C. G. Tseng

**Affiliations:** 1 TissueTech, Inc., Miami, FL, 33173, USA; 2 Department of Ophthalmology, Bascom Palmer Eye Institute, University of Miami Miller School of Medicine, Miami, FL, 33136, USA; 3 Flaum Eye Institute, University of Rochester Medical Center, Rochester, NY, 14642, USA; 4 Ocular Surface Center and Ocular Surface Research & Education Foundation, Miami, FL, 33173, USA

## Abstract

Proliferative vitreoretinopathy (PVR) is mediated by proliferation and epithelial mesenchymal transition (EMT) of retinal pigment epithelium (RPE). Because heavy chain-hyaluronic acid/pentraxin 3 (HC-HA/PTX3) purified from human amniotic membrane exerts anti-inflammatory and anti-scarring actions, we hypothesized that HC-HA/PTX3 could inhibit these PVR-related processes *in vitro*. In this study, we first optimized an ARPE-19 cell culture model to mimic PVR by defining cell density, growth factors, and cultivation time. Using this low cell density culture model and HA as a control, we tested effects of HC-HA/PTX3 on the cell viability (cytotoxicity), proliferation (EGF + FGF-2) and EMT (TGF-β1). Furthermore, we determined effects of HC-HA/PTX3 on cell migration (EGF + FGF-2 + TGF-β1) and collagen gel contraction (TGF-β1). We found both HA and HC-HA/PTX3 were not toxic to unstimulated RPE cells. Only HC-HA/PTX3 dose-dependently inhibited proliferation and EMT of stimulated RPE cells by down-regulating Wnt (β-catenin, LEF1) and TGF-β (Smad2/3, collagen type I, α-SMA) signaling, respectively. Additionally, HA and HC-HA/PTX3 inhibited migration but only HC-HA/PTX3 inhibited collagen gel contraction. These results suggest HC-HA/PTX3 is a non-toxic, potent inhibitor of proliferation and EMT of RPE *in vitro*, and HC-HA/PTX3’s ability to inhibit PVR formation warrants evaluation in an animal model.

Proliferative vitreoretinopathy (PVR) is characterized by membranes that develop on the surface of the retina in 5–10% of cases following rhegmatogenous retinal detachments^1^,^2^. Recurrent retinal detachments with PVR require additional surgical interventions and are associated with a poor visual outcome^3^. Prevention of PVR development during the initial retinal detachment repair could potentially improve the success rates and visual outcomes. Previous clinical studies have examined the use of triamcinolone acetonide^4^, heparin^5^, low molecular weight heparin^6^,^7^,^8^, 5-fluorouracil^6^,^7^,^8^, and daunorubicin^9^. However, none of these adjuvant pharmacologic therapies has shown consistent effectiveness in decreasing re-detachment rates or improving visual outcomes in patients with retinal detachments.

The main cellular components of PVR membranes are retinal pigment epithelial (RPE) cells^10^. A large body of evidence shows that vitreal growth factors and inflammatory cytokines drive the formation of PVR membranes^11^. Retinal breaks can result in RPE cells dispersing into the vitreous cavity where they are exposed to vitreous growth factors, such as transforming growth factor β (TGF-β) and inflammatory cytokines^12^,^13^,^14^. These growth factors and cytokines drive RPE cells to survive, proliferate, and undergo pathologic epithelial mesenchymal transition (EMT) in a non-physiologic environment^15^,^16^,^17^,^18^,^19^.

Amniotic membrane (AM) has advanced the treatment of inflammatory and disordered wound healing processes in ocular surface diseases such as corneal scars, burns, infections, autoimmune processes, and post-surgery, in part by regulating TGF-β signaling^20^,^21^. Studies have demonstrated that AMs inhibit several aspects of pro-inflammatory, pro-proliferative, and pro-EMT processes, including expression of TGF-β receptors I, II, and III^20^,^22^,^23^. Furthermore, AMs inhibit downstream TGF-β signaling cascade targets, which are essential for EMT, including expression of alpha smooth muscle actin (α-SMA), alternatively spliced domain A (EDA)-containing fibronectin (Fn), and integrin^20^,^22^,^23^. Additionally, AM extract can reverse TGF-β-induced myofibroblast differentiation *in vitro*^24^ and AM can suppress corneal opacities in a rabbit model^25^. Recently, we have successfully purified and characterized heavy chain-hyaluronic acid/pentraxin 3 (HC-HA/PTX3) from human AM as a unique matrix responsible for AM’s anti-inflammatory, anti-scarring and anti-angiogenic therapeutic actions^26^,^27^,^28^,^29^,^30^,^31^. As recently reviewed^32^, HC-HA/PTX3 is formed by tight association between pentraxin 3 (PTX3) and HC-HA, which consists of high molecular weight (HMW) hyaluronic acid (HA) covalently linked to heavy chain 1 (HC1) of inter-α-trypsin inhibitor (IαI) through the catalytic action of tumor necrosis factor-stimulated gene-6 (TSG-6). We have reported that HC-HA/PTX3 suppresses pro-inflammatory responses by inducing apoptosis of activated but not resting neutrophils (by fMLP or LPS) and macrophages (by LPS or/and IFN-γ)^29^; promoting polarization of M2 macrophages^29^; suppressing Th1 cells while promoting the expansion of regulatory T cells^30^. In addition, HC-HA/PTX3 inhibits scarring by downregulating mRNA expression of TGF-β1^27^ and protein expression of TGF-β1, TGFβRI, and TGFβRII in human corneal fibroblasts (our unpublished data). Furthermore, HC-HA/PTX3 suppresses HUVEC viability, proliferation and VEGF-induced tube formation^26^. Therefore, we would like to test the hypothesis that HC-HA/PTX3 can be an exciting potential biologic for preventing PVR formation.

## Results

### Optimization of an *in vitro* cell culture-based PVR model

Previously, we had established an *in vitro* cell culture-based model to discern the signaling pathways controlling proliferation and EMT, i.e., the two hallmarks of PVR, in human ARPE-19 cells^33^. In that model, post-confluent ARPE-19 cells were treated with EGTA before stimulation by epidermal growth factor (EGF) + fibroblast growth factor-2 (FGF-2) or EGF + FGF-2 + TGF-β1 to induce proliferation and EMT, respectively. However, we thought that this model might not fully recapitulate PVR conditions *in vivo* because RPE cells initially dispersed into the vitreous cavity are likely to be at a low density when they are exposed to a variety of growth factors and cytokines, including EGF, FGF-2, and TGF-β1.

**Figure 1:**
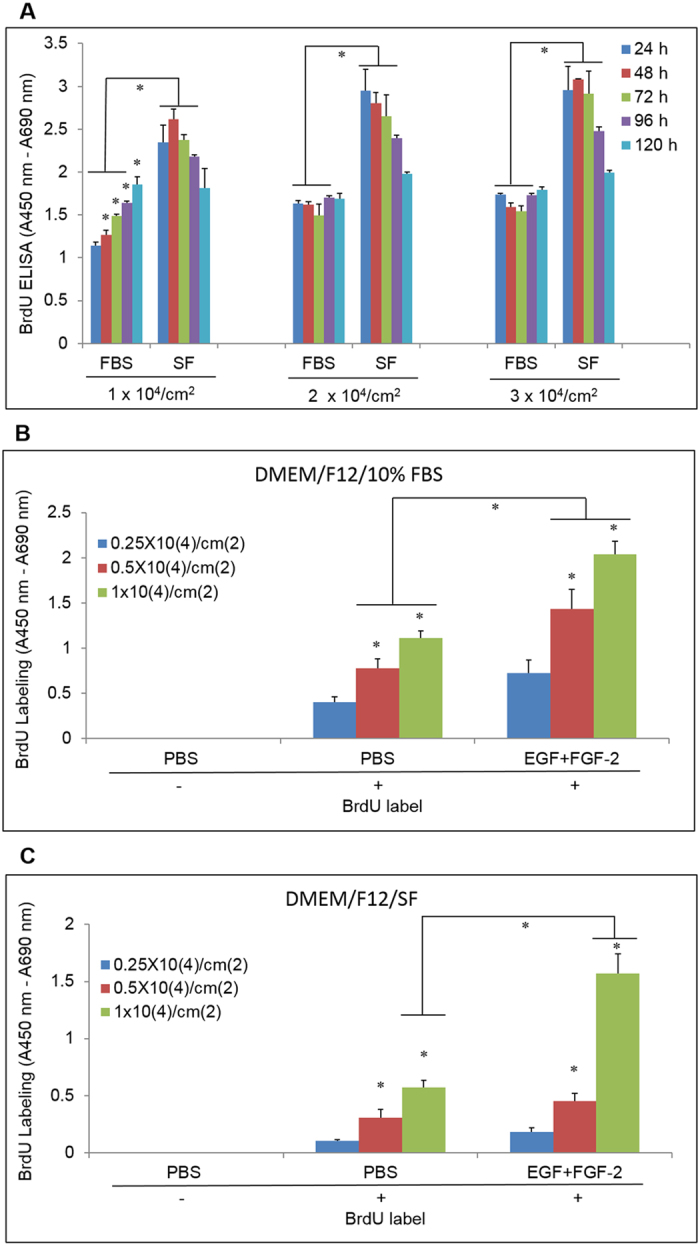
Proliferation affected by seeding density and culture time. (**A**) ARPE-19 cells in DMEM/F-12 medium with 10% FBS (FBS) or serum-free (SF) were seeded at 1 × 10^4^/cm^2^, 2 × 10^4^/cm^2^, and 3 × 10^4^/cm^2^ and incubated for 24–120 h. Proliferation was assayed with BrdU labeling (4 h) followed by BrdU ELISA. (**B**,**C**) The same as A except that cells were seeded at 0.25 × 10^4^/cm^2^, 0.5 × 10^4^/cm^2^, and 1 × 10^4^/cm^2^ in DMEM/F-12/10% FBS (**B**) or DMEM/F-12/SF (**C**) and stimulated with EGF (10 ng/ml) and FGF-2 (20 ng/ml) (EGF + FGF-2) for 48 h. (n = 4, *indicates p < 0.05 when compared at the same cultivation time (**A**) or compared with the lower density in the same treatment group or at the same density between groups treated with PBS and EGF + FGF-2 (**B**,**C**).

To modify this model^33^, we first examined the cell seeding density, the culture medium DMEM/F12 with 10% FBS or without FBS (serum free or SF), and the cultivation time to promote proliferation of ARPE-19 cells without EGF and FGF-2. We found that ARPE-19 cells at 1 × 10^4^/cm^2^ proliferated well, as measured by BrdU labeling, in the DMEM/F12/10% FBS during 24 h to 120 h of cultivation. In contrast, cells seeded at higher density of 2 × 10^4^/cm^2^ and 3 × 10^4^/cm^2^ barely proliferated during the same period (Fig. 1A), likely due to cell contact inhibition. Unexpectedly, ARPE-19 cells in DMEM/F12/SF proliferated more than those in DMEM/F12/10% FBS (Fig. 1A). This unusual phenomenon had also been reported in other studies^34^,^35^. Jun *et al*.^34^ showed serum starvation of ARPE-19 cells for 24 hours caused rapid induction of c-met and HGF mRNA and protein expression. ARPE-19 cell proliferation was also enhanced with recombinant HGF treatment. Neutralization against c-Met inhibited the proliferation of serum-deprived ARPE-19 by 64.5% (n = 9, S.D. 5.5%). Therefore, this unusual proliferation might be attributed to the activation of HGF and its receptor c-Met during serum starvation. However, we found the proliferation in ARPE-19 cells in the DMEM/F12/SF at all three cell seeding densities decreased during 72 h to 120 h of cultivation, suggesting that prolonged serum starvation might induce cell death or differentiation.

We then tested the proliferation of ARPE-19 cells under even lower cell seeding densities (0.25 × 10^4^/cm^2^, 0.5 × 10^4^/cm^2^, and 1 × 10^4^/cm^2^) and a shortened cultivation period (48 h) in DMEM/F12/10% FBS or DMEM/F12/SF and stimulated by EGF + FGF-2. We found that the cell proliferation measured by BrdU labeling in DMEM/F12/10% FBS induced by EGF (10 ng/ml) and FGF-2 (20 ng/ml) was cell density-dependent. The proliferation was significantly induced by EGF + FGF-2 at 0.5 × 10^4^/cm^2^ and 1 × 10^4^/cm^2^ (p = 0.02 and 0.002, respectively) but not at 0.25 × 10^4^/cm^2^ (p = 0.07) when compared to the PBS control (Fig. 1B). Between the cell seeding densities of 0.5 × 10^4^/cm^2^ and 1 × 10^4^/cm^2^, the proliferation induced by EGF + FGF was about the same (83.7 ± 1.5% vs 83.9 ± 1.0%). In the DMEM/F12/SF, proliferation was only significantly induced by EGF + FGF-2 at 1 × 10^4^/cm^2^ (p = 0.005) (Fig. 1C). For consistency, we chose 1 × 10^4^/cm^2^ and DMEM/F12/10% FBS for the remaining experiments, since these culture conditions led to the largest amount of ARPE-19 cell proliferation.

**Figure 2:**
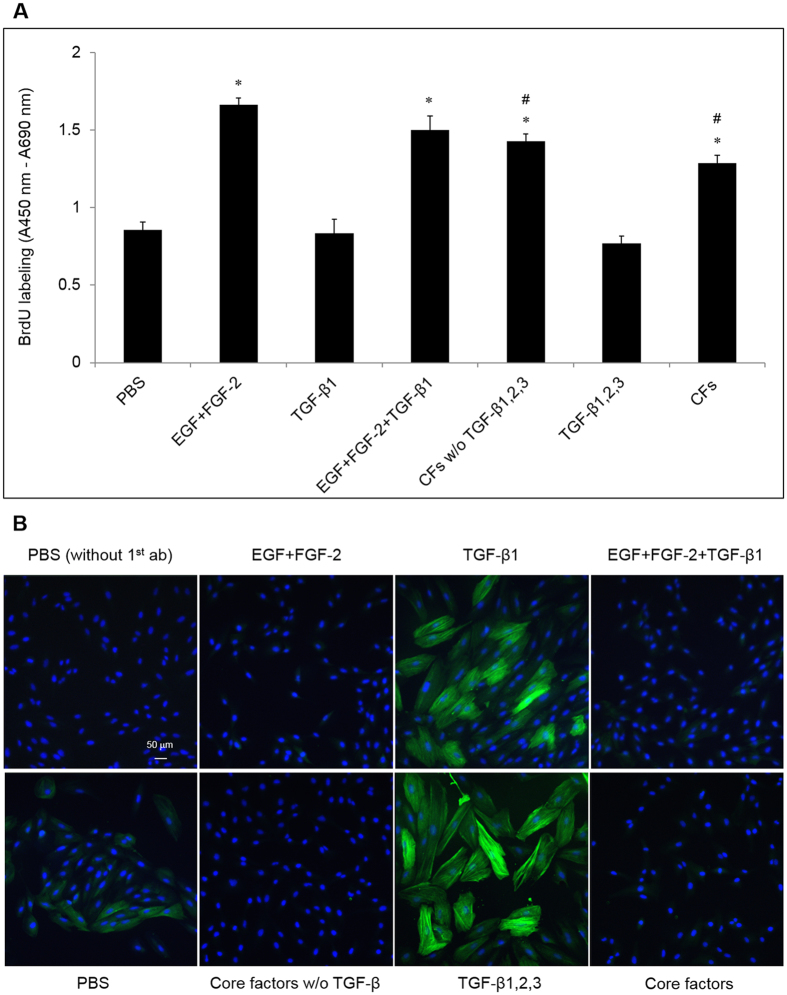
Proliferation and EMT affected by EGF + FGF-2 + TGF-β1 and the “core factors” (CFs). (**A**) BrdU labeling (**A**, n = 3, *indicates p < 0.05 compared to the PBS control and # indicates p < 0.05 compared to EGF + FGF-2) and immunostaining α-SMA (**B**, nuclear counterstaining with Hoechst 33342, scale bar = 50 μm) of ARPE-19 cells seeded at 1 × 10^4^/cm^2^ in DMEM/F12/10% FBS for 24 h and then treated with PBS or EGF (10 ng/ml) + FGF-2 (20 ng/ml) (EGF + FGF-2), TGF-β1, β2, β3 (each at 10 ng/ml), or CFs (see Table 1) for 48 h.

Next, we compared the ability of EGF + FGF-2 with that by the “core factors (CFs)” (18 growth factors and cytokines [Table 1] which have been identified in the vitreous samples of PVR patients and demonstrated to induce PVR when injected intravitreally into normal rabbits^11^,^18^) in promoting proliferation of ARPE-19 cells. We found EGF + FGF-2, EGF + FGF-2 + TGF-β1, the “CFs” without (w/o) TGF-β1, 2, 3, and the “CFs” all induced significant proliferation in ARPE-19 cells when compared with that with PBS (Fig. 2A, p = 8.1 × 10^−5^, 0.003, 0.0003, 0.001, respectively, and *indicates p < 0.05). Among these, EGF + FGF-2 induced higher proliferation than that by the “CFs” w/o TGF-βs and “CFs” (p = 0.002 and 0.0001, respectively, and # also indicates p < 0.05, Fig. 2A), but not that by EGF + FGF-2 + TGF-β1 (p > 0.05). In addition, TGF-β1 or TGF-β1, 2, 3 did not significantly inhibit EGF + FGF-2-induced proliferation of ARPE-19 cells (p > 0.05). These results show that EGF + FGF-2 induced even more proliferation in ARPE-19 cells *in vitro* than the “CFs”.

Finally, we compared the ability of TGF-βs and the “CFs” to induce EMT. There was a weak but positive α-SMA immunostaining detected in ARPE-19 cells cultured in DMEM/F12/10% FBS (Fig. 2B). However, much stronger staining of α-SMA was induced by TGF-β1 (10 ng/ml). Even stronger staining of α-SMA was seen with the addition of TGF-β2 (10 ng/ml) and TGF-β3 (10 ng/ml). In contrast, EGF + FGF-2 dramatically inhibited the expression of α-SMA induced by TGF-β1; the “CFs” with or without TGF-β did not effectively induce α-SMA expression.

Taken together, we have modified our previous cell culture-based model to induce proliferation and EMT in ARPE-19 cells at a low density (1 × 10^4^/cm^2^) in DMEM/F12/10% FBS under the stimulation with EGF + FGF-2 and TGF-β1 (or together with TGF-β2 and TGF-β3), respectively, for as short as 48 h.

### Biochemical analysis of HC-HA/PTX3 purified from human AM

**Figure 3:**
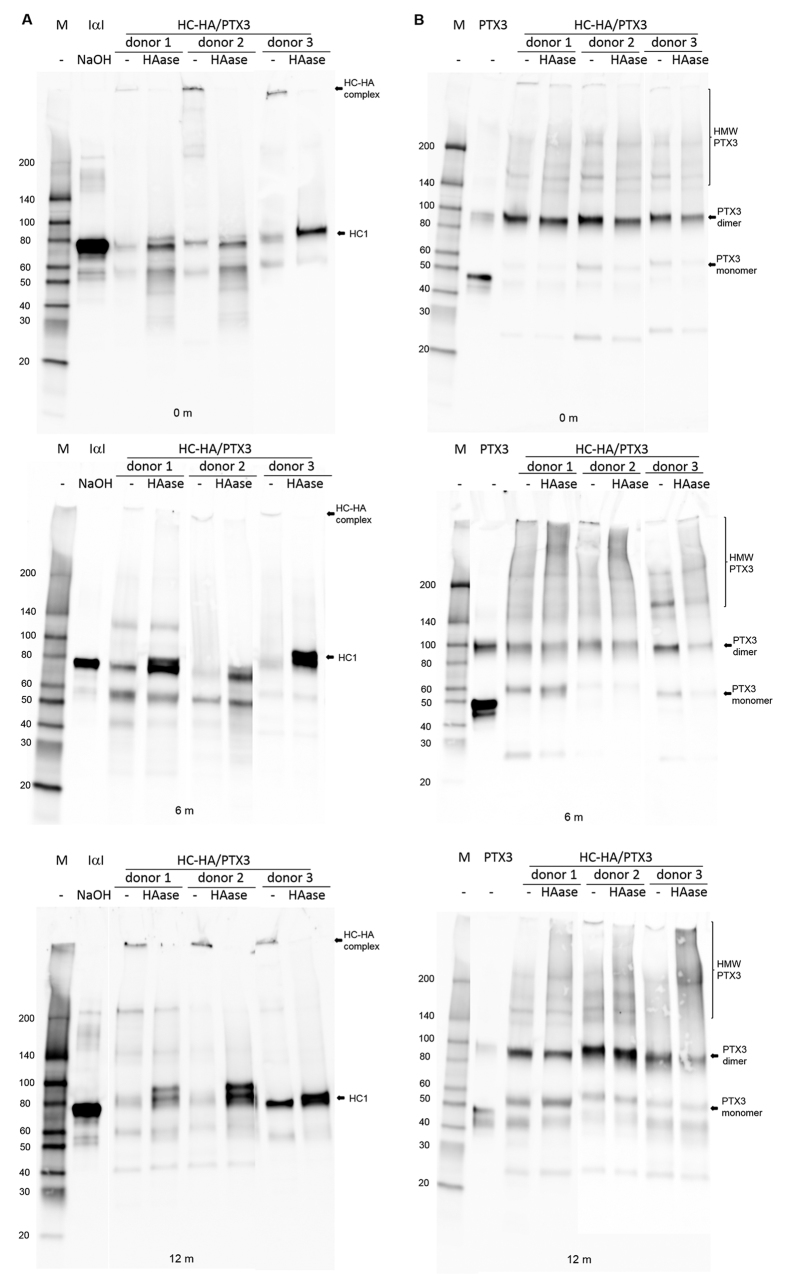
The biochemical characterization of purified HC-HA/PTX3. HC-HA/PTX3 purified from three donor lots (10 μg of HA each) was digested without (−) or with (+) hyaluronidase (1 U/μg HA) at 37 °C for 1 h and analyzed by Western blot for HC1 (**A**, using purified human IαI as the control that released HC1 after being treated with 100 mM NaOH at ~25 °C for 1 h), and PTX3 (**B**, using human recombinant PTX3 as the control). For examining the stability, lyophilized HC-HA/PTX3 was stored at −80 °C for 6 months (6 m) and 12 months (12 m), followed by Western blot (as described above) to check the integrity of HC1 (**A**) and PTX3 (**B**). Note: for the clarity, some unused blank lanes were cut off. The full images of all blots were present and exposure (brightness and contrast) was applied to the whole image.

To demonstrate the reproducible manufacturing process of HC-HA/PTX3, we had prepared HC-HA/PTX3 from three lots of human AM according to our published method^27^ under the Good Laboratory Practice (GLP) standard. The content of HA in each lot of HC-HA/PTX3 was 19.6 μg/ml, 21.6 μg/ml, and 26.0 μg/ml, respectively (22.4 ± 2.7 μg/ml) with HMW HA size (>4000 kDa, data not shown) while the total protein in each lot of HC-HA/PTX3 was below the detection limit (25 μg/ml), in agreement with our previous report^27^. Western blot analysis showed both HC1 and PTX3 were present in each lot and HC1 was released after the digestion with hyaluronidase (HAase), confirming their identity of freshly prepared HC-HA/PTX3 in two weeks designated as 0 month (Fig. 3A and B). After being stored at −80 °C for about six months (6 m) and 12 months (12 m) in a lyophilized form, the identity of this HC-HA/PTX3 did not change (Fig. 3A and B), indicating the stability of HC-HA/PTX3 was at least 12 months. These three lots of HC-HA/PTX3 were used within a 12 month period in the remaining experiments.

### HC-HA/PTX3 but not HA specifically and dose-dependently inhibits proliferation of RPE cells induced by EGF and FGF-2

**Figure 4:**
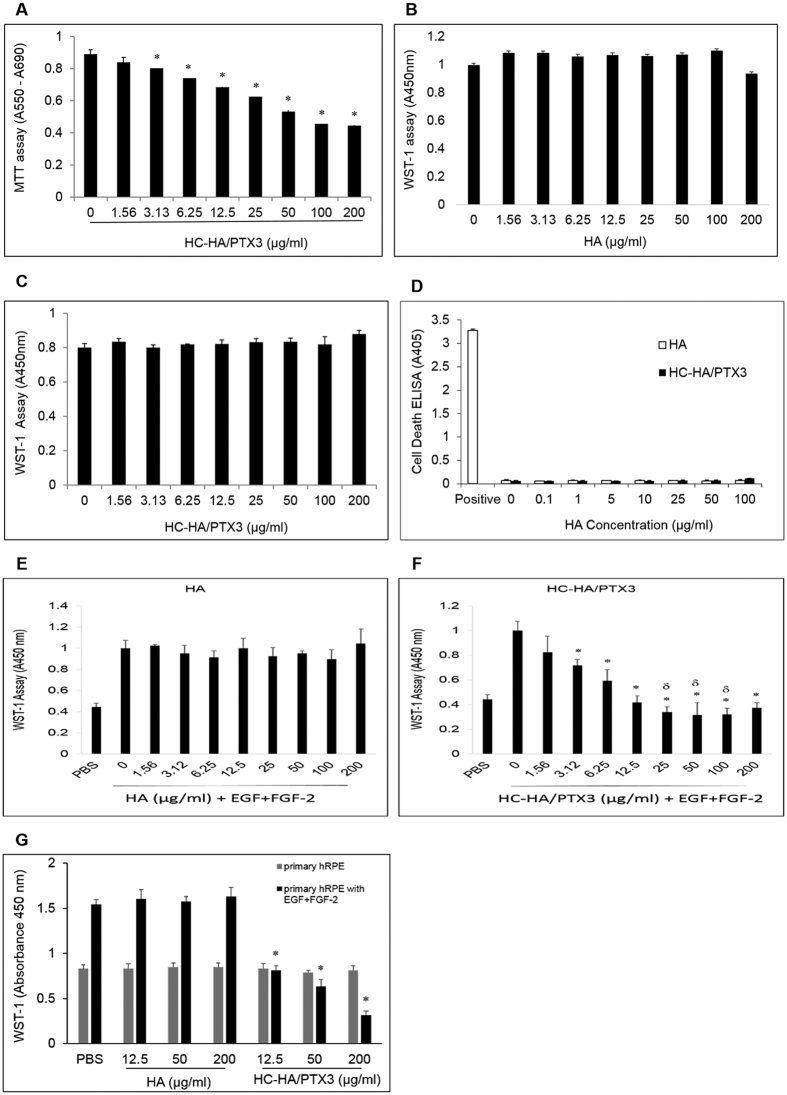
Cytotoxicity and proliferation measured by MTT and WST-1. Cytotoxicity- ARPE-19 cells seeded at 1 × 10^4^/cm^2^ were treated with an increasing doses of HC-HA/PTX3 or HA for 48 h before being measured by MTT (**A**), WST-1 (**B**,**C**), or cell death detection ELISA (**D**). Proliferation - In a separate experiment, ARPE-19 cells (**E**,**F**) or primary human RPE cells (**G**) were seeded and treated similarly as in cytotoxicity except the cells were also stimulated by EGF (10 ng/ml) and FGF-2 (20 ng/ml) (n = 3, *indicates p < 0.05 compared with the PBS control).

We first determined whether HC-HA/PTX3 (1.56 to 200 μg/ml) was toxic to ARPE-19 cells (without stimulation by EGF and FGF-2) by the MTT assay. Microscopic observation did not reveal any noticeable morphological changes or cell death after HC-HA/PTX3 treatment for 24 h–48 h when compared to the PBS control. However, the MTT assay indicated a severe cytotoxicity by HC-HA/PTX3 starting at 3.13 μg/ml (Fig. 4A), which was correlated with a dramatic increase of needle-like crystals in cells (data not shown). MTT is membrane impermeable and taken up by cells through endocytosis. Reduced MTT formazan accumulates in the endosomal/lysosomal compartment before being transported to the cell surface through exocytosis^37^ to generate needle-like formazan crystals^38^. Such endocytosis of MTT does not cause obvious lesions but induces cell death^39^. So, we speculate that HC-HA/PTX3 might enhance the endocytosis of MTT to cause this artifact of cytotoxicity. To overcome this pitfall, we then chose WST-1 assay because, unlike MTT, WST-1 is converted to a *soluble* formazan and diffuses outside of cells freely. Our repeated experiments confirmed no cytotoxicity by HA and HC-HA/PTX3 (up to 200 μg/ml) in unstimulated ARPE-19 cells (Fig. 4B and C). To further confirm this result, we performed the additional test using a Cell Death Detection ELISA (Roche, cat# 11544675001), which determines histone-associated DNA fragments generated by cell death. Cell lysates of normal ARPE-19 cells (e.g., not stimulated by EGF, FGF-2, or TGF-β1) after 48-hour treatment with a series of HA or HC-HA/PTX3 were collected separately and assayed. The data shows that both HA (0–100 μg/ml) and HC-HA/PTX3 (0–100 μg/ml) do not cause cell death of normal ARPE-19 cells (Fig. 4D). In addition, we also tested the cytotoxicity of HC-HA/PTX3 in a rabbit PVR model by intravitreal injection of 0.1 ml of HC-HA/PTX3 (25 μg/ml, 50 μg/ml, or 75 μg/ml) into each eye. Both weekly electroretinography (ERG) and fundus monitor (for 4 weeks) did not show any abnormal effect in HC-HA/PTX3 treatment groups when compared with PBS treatment group. Histopathology results also confirmed this finding (Kuriyan *et al*., ARVO 2016, poster number:1126). Therefore, *in vitro* and *in vivo* studies have shown that HC-HA/PTX3 is not toxic to normal RPE cells.

Because BrdU labeling could not accurately measure proliferation when cells reached a high density (Fig. 1A), we thus examined whether the WST-1 assay could overcome this limitation. When ARPE-19 cells were seeded at non-confluent cell densities, i.e., from 0.03125 × 10^4^/cm^2^ to 2 × 10^4^/cm^2^, both WST-1 assay (R^2^ = 0.9986) and BrdU ELISA (R^2^ = 0.9591) gave a good linear relationship. In contrast, when ARPE-19 cells were seeded at the confluent cell density (4 × 10^4^/cm^2^), the WST-1 assay (R^2^ = 0.9721) was superior to BrdU ELISA (R^2^ = 0.8429) because of its good linearity. Therefore, we used the WST-1 assay to measure cell proliferation thereafter. In doing so, we found that HC-HA/PTX3 (Fig. 4E) but not HA (Fig. 4F), starting from 3.13 μg/ml, significantly inhibited proliferation of ARPE-19 cells induced by EGF (10 ng/ml) and FGF-2 (20 ng/ml) compared with the cells treated with PBS, EGF and FGF-2 (p < 0.05 and indicated by*). The finding that HC-HA/PTX3 was not toxic to unstimulated RPE cells but inhibited proliferation of RPE cells under stimulation of EGF + FGF-2 was also verified in primary human RPE cells (Fig. 4G).

### HC-HA/PTX3 inhibits migration induced by EGF, FGF-2, and TGF-β1 and collagen gel contraction induced by TGF-β1

**Figure 5:**
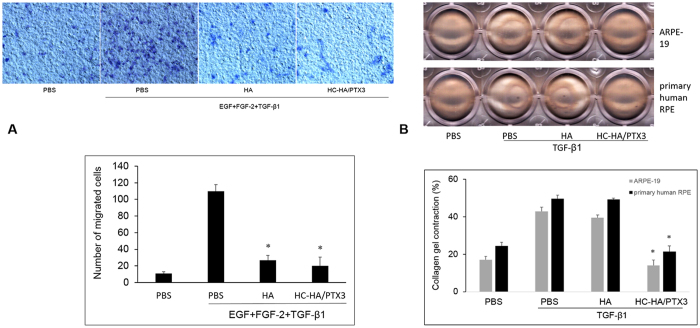
Inhibition of RPE cell migration and collagen gel contraction by HC-HA/PTX3. (**A**) (migration). ARPE-19 cells (2 × 10^6^/ml) treated with PBS (vehicle control), HA (25 μg/ml), or HC-HA/PTX3 (25 μg/ml) were in the upper compartment while DMEM/F12 without or with EGF (10 ng/ml), FGF-2 (20 ng/ml), and TGF-β1 (10 ng/ml) were in the lower compartment of transwell wells. After incubation for 8 h, the number of migrated cells were counted from six random microscopic fields (n = 4, *indicates p < 0.05 when compared with PBS + EGF + FGF-2 + TGF-β1). (**B**) (collagen gel contraction). ARPE-19 cells or primary human RPE cells (5 × 10^5^/ml) without or with TGF-β1 (10 ng/ml) and treated with PBS (vehicle control), HA (25 μg/ml), or HC-HA/PTX3 (25 μg/ml) were on the top of collagen gel. The percentage of gel contraction was determined by measuring the gel size at 72 h when compared to the initial size (at 0 h) in each group (n = 4, * indicates p < 0.05 compared with PBS + TGF-β1).

RPE cells migrate during EMT^40^,^41^ and participate in contraction^42^,^43^ of epiretinal membranes^11^,^44^. We thus examined these two PVR-related cell behaviors *in vitro*. Our results showed that HC-HA/PTX3 (25 μg/ml) as well as HA (25 μg/ml) completely suppressed the migration of ARPE-19 cells under stimulation by EGF (10 ng/ml), FGF-2 (20 ng/ml), and TGF-β1 (10 ng/ml) (Fig. 5A). In contrast, HC-HA/PTX3, but not HA, significantly reduced the TGF-β1-induced collagen gel contraction in both ARPE-19 cells and primary human RPE cells (Fig. 5B).

### HC-HA/PTX3 down-regulates Wnt/β-catenin and TGF-β1/Smad2/3 signaling

**Figure 6:**
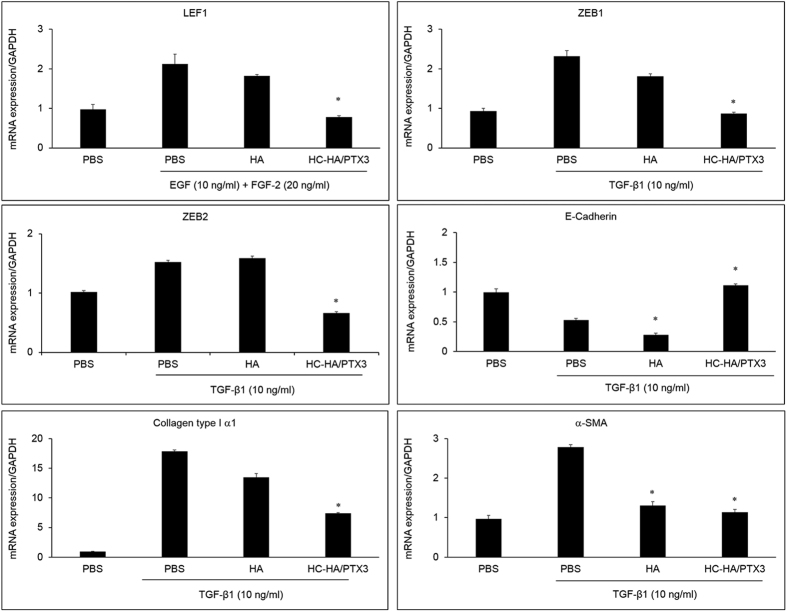
Suppression of mRNA expression of key markers in Wnt/β-catenin and Smad2/3 signaling by HC-HA/PTX3. ARPE-19 cells seeded at 1 × 10^4^/cm^2^ were simultaneously treated with EGF (10 ng/ml) and FGF-2 (20 ng/ml) or TGF-β1 (10 ng/ml) together with PBS, HA (25 μg/ml), or HC-HA/PTX3 (25 μg/ml) for 4 h. The mRNA expression was measured by the real-time PCR assay. n = 4, *indicates p < 0.05 vs. PBS + EGF + FGF-2 or PBS + TGF-β1.

**Figure 7:**
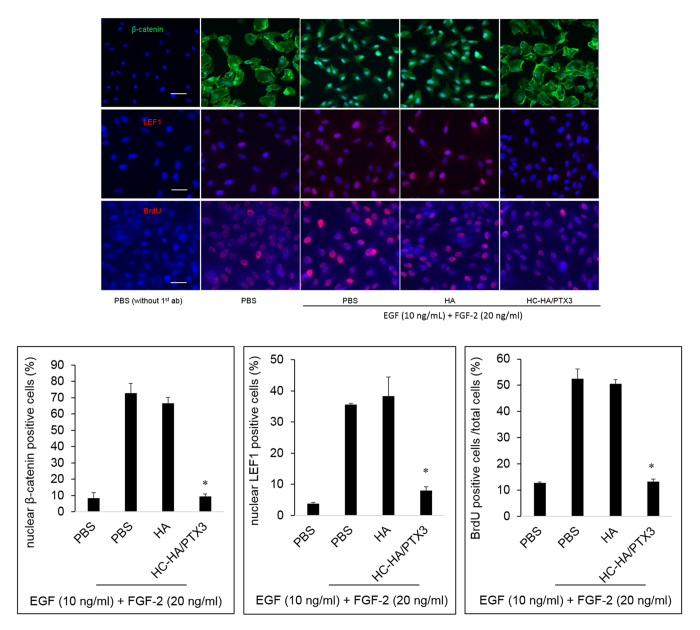
Suppression of nuclear localization of β-catenin and LEF1 by HC-HA/PTX3. ARPE-19 cells seeded at 1 × 10^4^/cm^2^ were simultaneously treated with EGF (10 ng/ml) and FGF-2 (20 ng/ml) together with PBS, HA (25 μg/ml), or HC-HA/PTX3 (25 μg/ml) for 24 h (for β-catenin and LEF1) or 48 h (for BrdU). The expression and nuclear localization of β-catenin, LEF1, and BrdU was detected by immunostaining. Nuclei were counterstained by Hoechst 33342. The scale bar = 50 μm, n = 4, *indicates p < 0.05 vs. PBS + EGF + FGF-2.

In a post-confluent cell culture-based model, proliferation and EMT in ARPE-19 cells are mainly regulated by Wnt/β-catenin signaling and TGF-β1/Smad2/3 signaling, respectively^33^. Herein, we noted that the same signaling was also activated in the low cell density model. As shown in Fig. 2, EGF and FGF-2 induced proliferation of ARPE-19 cells, while TGF-β1 induced EMT. To further discern the signaling pathway controlling proliferation induced by EGF and FGF-2, we stimulated ARPE-19 cells simultaneously with EGF and FGF-2 and treated with 25 μg/ml HMW HA or 25 μg/ml HC-HA/PTX3 for 48 h, in the aforementioned optimized *in vitro* model. We found that the mRNA expression of lymphoid enhancer factor 1 (LEF1), which acts downstream in Wnt signaling and binds to Wnt response elements to provide docking sites for β-catenin^45^, was significantly up-regulated by EGF and FGF-2. The EGF and FGF-2-induced up-regulation was unaffected by HA, but significantly down-regulated by HC-HA/PTX3 (Fig. 6), although the mRNA expression of β-catenin itself was not significantly changed (data not shown). Furthermore, the immunostaining data showed that EGF and FGF-2 induced the nuclear localization of β-catenin and LEF1 (Fig. 7), signifying the activation of canonical Wnt signaling. However, HC-HA/PTX3, but not HA, significantly suppressed the nuclear localization of β-catenin and LEF1 (Fig. 7). As expected, the immunostaining data also showed a significant increase in BrdU labeling by EGF and FGF-2, which was significantly reduced by HC-HA/PTX3 but not HA, in ARPE-19 cells (Fig. 7). These data support that the inhibition of EGF and FGF-2-induced proliferation of ARPE-19 cells by HC-HA/PTX3 was mediated by down-regulation of canonical Wnt/β-catenin signaling.

**Figure 8:**
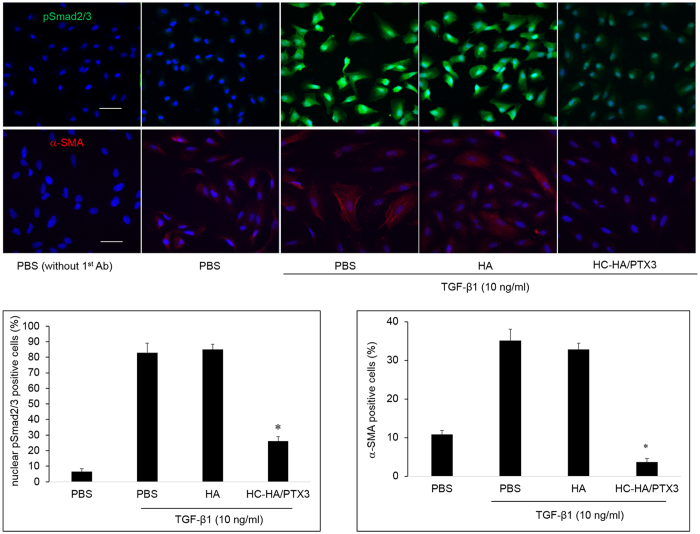
Suppression of nuclear localization of pSmad2/3 and α-SMA expression by HC-HA/PTX3. ARPE-19 cells seeded at 1 × 10^4^/cm^2^ were simultaneously treated with TGF-β1 (10 ng/ml) together with PBS, HA (25 μg/ml), or HC-HA/PTX3 (25 μg/ml) for 2 h (for pSmad2/3) and 48 h (for α-SMA). The nuclear localization of pSmad2/3 and expression of α-SMA was detected by immunostaining. Nuclei were counterstained by Hoechst 33342. The scale bar = 50 μm, n = 4, *indicates p < 0.05 vs. PBS + TGF-β1.

To examine the signaling pathway controlling the EMT induced by TGF-β1, ARPE-19 cells were simultaneously stimulated with TGF-β1 and treated with 25 μg/ml HMW HA or 25 μg/ml HC-HA/PTX3, for 2 h (for pSmad2/3) or 48 h (for α-SMA). We found that the mRNA expression of ZEB1 but not ZEB2 (both transcriptional factors acting downstream in the TGF-β1/Smad2/3 signaling)^46^ was significantly up-regulated by TGF-β1, which was significantly down-regulated by HA and HC-HA/PTX3 (Fig. 6). Similarly, the mRNA expression of collagen type I α1 and α-SMA was up-regulated by TGF-β1 but down-regulated by HC-HA/PTX3. Downregulation of E-cadherin, one of the hallmarks of EMT^47^, was observed by TGF-β1 stimulation and further enhanced by HA, but dramatically reversed by HC-HA/PTX3 (Fig. 6). In addition, the immunostaining data showed that TGF-β1 induced the strong nuclear localization of pSmad2/3, while HC-HA/PTX3, but not HA, significantly suppressed the nuclear localization of pSmad2/3 (Fig. 8). As expected, the expression of α-SMA was also down-regulated by HC-HA/PTX3, but not by HA (Fig. 8). These results suggested that suppression of EMT by HC-HA/PTX3 was through suppression of nuclear translocation of pSmad2/3.

## Discussion

The development of PVR is critically dependent on growth factors and cytokines present in the pathological milieu, which collectively prolong survival^48^, proliferation^18^,^33^, and EMT^49^,^50^,^51^,^52^ of dispersed RPE cells in the vitreous cavity. Among them, TGF-β is critical in promoting EMT, migration of RPE cells, and PVR membrane contraction^50^,^53^,^54^. Consistent with our previous report using a post-confluent (high density) cell culture-based model^33^, we herein demonstrated that both proliferation and EMT, i.e., the two pathogenic hallmarks of PVR, of ARPE-19 cells, can be promoted by EGF + FGF-2 and TGF-β1 via Wnt/β-catenin and TGF-β/Smad2/3 signaling, respectively, in an optimized low density cell culture model in an FBS-containing medium (Figs 1 and 2). In this model, we noted that proliferation induced by EGF + FGF-2 was higher than that by “CFs”, a finding that might be explained by the fact that the “CFs” includes IFN-γ which had been reported to inhibit proliferation in human fetal RPE cells stimulated by PDGF-BB, -CC, and –DD, as well as to induce apoptosis^55^. We also noted that EGF + FGF-2 dramatically suppressed the expression of α-SMA induced by TGF-β1 in the FBS-containing medium (Fig. 2B). This result differed from what we have reported using post-confluent high density cultures^33^, suggesting that such expression of α-SMA is greatly influenced by the cell density^56^. We think that the optimized cell model is better at mimicking the *in vivo* development of PVR where RPE cells are dispersed in the vitreous cavity at low densities initially and driven to proliferate by EGF and FGF-2. Subsequently, at a higher density, the RPE cells are driven to EMT. Using this optimized model, we noted that HC-HA/PTX3, but not HA, inhibits both proliferation and EMT of RPE cells, as well as suppresses cell migration and collagen gel contraction (Figs 5, 6, 7 and 8). Importantly, HC-HA/PTX3 did not affect the viability of RPE cells in the absence of EGF + FGF-2 (Fig. 4C and D). This finding is consistent with our previous reports that HC-HA/PTX3 specifically suppresses activated but not resting neutrophils^27^,^29^,^57^, macrophages^29^, and lymphocytes^27^. The cytotoxicity of HC-HA/PTX3 demonstrated by the MTT assay (Fig. 4A and B) but not the WST-1 assay was likely due to enhanced exocytosis of insoluble MTT formazan from cells by HC-HA/PTX3, leading to cell damage^37^,^39^. The same phenomenon of cytotoxicity that we observed herein has also been reported for amyloid β peptide^39^,^58^ and mesoporous silica nanoparticles^59^ when both MTT and WST-1 assays were compared. Further pursuit of this putative property of HC-HA/PTX3 may help us disclose additional mechanistic insight into the actions mediated by HC-HA/PTX3.

Inasmuch as we have gained significant understanding of the pathogenesis of PVR during the past decades, the surgical management of this disease is still unsatisfactory. While anti-inflammatory agents, such as corticosteroids, ameliorate PVR in animal models^60^,^61^, they have not been efficacious in human trials^62^. Other anti-proliferative agents (5-fluorouracil^63^, daunorubicin^9^, 5-fluorouracil with low molecular weight heparin^6^,^8^,^64^, and DNA-RNA chimeric ribozyme^65^), growth factor pathway inhibitors (hypericin, an inhibitor of protein kinase C^66^ and tranilast, an inhibitor of TGF-β1^67^) and antioxidant agents (N-acetylcysteine^68^) have failed to either decrease re-detachment rates or improve visual outcomes. These unsuccessful outcomes suggest that the pathogenesis of PVR is multifactorial and complex. Indeed, immunohistochemical studies have revealed that at least RPE cells, fibroblasts, and macrophages are present in PVR membranes^11^,^69^,^70^. Other cells found in PVR membranes include Müller/glial cells, fibroblasts/myofibroblasts, and a myriad of immune cells (macrophages, monocytes, T lymphocytes, B lymphocytes, and cells expressing HLA-DR and DQ)^71^. Injection of each individual type of these cells also induces PVR in animal models^72^,^73^,^74^. It is no wonder therapeutic options that target only one factor or a single cellular process have not been effective^62^,^75^.

Consequently, HC-HA/PTX3 purified from AM is a potential novel biologic that can help address such an unmet clinical need by circumventing the aforementioned shortcomings. As demonstrated herein, HC-HA/PTX3 uniquely suppresses proliferation, EMT, migration, and gel contraction mediated by RPE cells. As reviewed by Tseng^76^, HC-HA/PTX3’s anti-inflammatory action applies to activated but not resting neutrophils^27^,^29^,^57^, macrophages^29^, and lymphocytes^27^, i.e., extending from innate to adaptive immune responses. In addition, its anti-scarring action applies to human corneal fibroblasts to downregulate TGF-β1 promoter activity^30^ and its anti-angiogenic action applies to human umbilical vascular endothelial cells to inhibit cell viability, proliferation, migration, and tube formation^26^. In animals, we have reported that subconjunctival injection of HC-HA/PTX3 down-regulates activation of CD4^+^ Th1 cells to prolong the survival of murine corneal allografts^30^ and reduces infiltration of bone marrow-derived fibrocytes and immune cells to the lacrimal glands and conjunctiva to avert dry eye in chronic graft-versus-host disease (cGVHD) (Scientific Reports 7, Article number: 42195 (2017) doi:10.1038/srep42195). Now that we have gathered strong *in vitro* data supporting the safety and efficacy of HC-HA/PTX3 in suppressing multiple pathological processes of RPE cells, we are well poised to test it in our established rabbit PVR model (Kuriyan *et al*., ARVO 2015, poster number: 2287).

## Materials and Methods

### Materials

Human ARPE-19 cell line and primary RPE cells were from ATCC (Manassas, VA) and Lonza (Walkersville, MD), respectively. Dulbecco’s Modified Eagle’s medium (DMEM), Ham’s/F12 medium, HEPES buffer, phosphate-buffered saline (PBS), penicillin-streptomycin, fetal bovine serum (FBS), 0.05% trypsin/EDTA, recombinant human CTGF, and Alexa Fluor-conjugated secondary IgG were from Invitrogen (Carlsbad, CA, USA). Recombinant human EGF, FGF-2, HGF, IGF-1, IL-6, PDGF-AA, PDGF-AB, PDGF-BB, PDGF-CC, PTX3, and VEGF were from R & D Systems (Minneapolis, MN). Recombinant human G-CSF, IFN-γ, MCP-1, TGF-α, TGF-β1, TGF-β2, and TGF-β3 were from PeproTech (Rocky Hill, NJ). Hyaluronidase, protease inhibitors, bovine serum albumin (BSA), paraformaldehyde, methanol, Triton X-100, and Hoechst 33342 dye were from Sigma (St Louis, MO). α-SMA and BrdU antibodies were from Abcam (La Jolla, CA); β-catenin antibody was from BD Biosciences (San Jose, CA); Antibodies to lymphoid enhancer factor 1 (LEF1) and phospho-Smad2/3 were from Cell Signaling Technology (Danvers, MA). HMW HA, Healon^®^ (~4,000 kDa, medical grade), was from Advanced Medical Optics (Santa Ana, CA). BCA Protein Assay Kit was from Pierce (Rockford, IL). HA quantitation kit was from Corgenix (Broomfield, CO). Plastic culture dishes were from Becton Dickinson (Lincoln Park, NJ).

## Methods

### Purification of HC-HA/PTX3 from Human AM

As reported^27^, HC-HA/PTX3 was prepared from cryopreserved human placentas^77^ provided by Bio-Tissue, Inc. (Miami, FL). AM from the same donor was extracted by PBS (pH 7.4) to generate PBS extract as reported^27^. The extract was then fractionated by ultracentrifugation in a CsCl gradient at an initial density of 1.35 g/ml in 4 M GnHCl at 35,000 rpm for 48 h at 15 °C (Optima™ L-80 X, SW41 rotor, Beckman Coulter, Indianapolis, IN). A total of 12 fractions (1 ml/fraction) was collected from each ultracentrifuge tube. The weight of each fraction was measured to calculate the density. After the biochemical analysis (HA ELISA and BCA protein assay, see below), fractions containing HA but little or no proteins were pooled and subjected to the second run of ultracentrifugation in a CsCl gradient at an initial density of 1.40 g/ml^27^. Selective fractions (containing HA but undetectable proteins) designated as HC-HA/PTX3 were pooled and dialyzed against distilled water, lyophilized, and stored at −80 °C. Therefore, the amount of HC-HA/PTX3 was expressed based on the HA amount present in the complex.

### Biochemical Characterization of HC-HA/PTX3

As reported^27^,^29^, the content of HA and proteins were measured by the enzyme-linked immunosorbent HA Quantitative Test Kit (Corgenix, Broomfield CO) and the BCA Protein Assay Kit (Life Technologies, Grand Island, NY), respectively. These two methods have been validated by our R & D Department. Western blotting was used to detect the presence of HC1 (ab70048, abcam, Cambridge, MA) and PTX3 (ALX-804-464-C100, Enzo Life Sciences, Farmingdale, NY) in purified HC-HA/PTX3 with or without HAase digestion (1 U/μg HA with supplement with protease inhibitors, Sigma-Aldrich, St. Louis, MO).

### Western Blotting

Samples were electrophoresed on 4–15% (w/v) gradient acrylamide ready gels (Bio-Rad, Hercules, CA) under denaturing and reducing conditions, and transferred to a nitrocellulose membrane (Bio-Rad), which was then blocked with 5% (w/v) fat-free milk powder in Tris-buffered saline (10 mM Tris-HCl, pH 7.4,150 mM NaCl, TBS) with 0.05% Tween-20 (TBST) for 1 h at room temperature, and washed with TBST three times for 10 min each. Then, specific primary antibodies were incubated with the membrane at 4 °C overnight. After three washes with TBST, the membranes were incubated with horseradish peroxidase-conjugated secondary antibodies diluted in TBST for 1 h. The membranes were then washed with TBST three times for 10 min each followed by TBS once for 5 min, and immunoreactive proteins were detected with Western Lighting Chemiluminescence Reagent (Perkin-Elmer, Waltham, MA, USA) and images captured by GE ImageQuant CAS 4000 (GE Healthcare Bio-Sciences, Pittsburgh, PA).

### Cell Culture and Treatment

ARPE-19, a human diploid RPE cell line, was cultured in HEPES-buffered DMEM and Ham’s F-12 (1:1) supplemented with 10% FBS, 50 units/ml penicillin, and 50 μg/ml streptomycin at 37 °C in humidified air with 5% CO_2_. For post-confluence experiments, cells were continuously cultured for 7 days upon 100% confluence before being tested. For low cell density assays, cells were seeded at 1 × 10^4^/cm^2^ or other densities overnight (20–24 h) followed by treatment with growth factors and cytokines for 24–120 h or 48 h (after optimization). In the case of serum starvation, cells were incubated in serum-free (SF) medium for 24 h followed by treatment with growth factors and cytokines for 24–120 h. BrdU (10 μM) labeling was performed for 4 h prior to the termination of the growth factors/cytokines treatment.

### Cell Viability and Proliferation

MTT and WST-1 assays were used to measure the cell viability while BrdU ELISA and WST-1 assays were used to measure the cell proliferation. For the MTT assay, ARPE-19 cells were seeded at 1 × 10^4^/cm^2^ in 96-well plate in DMEM/F12/10% FBS for 24 h and simultaneously treated with PBS (vehicle control), HA, or HC-HA/PTX3 of different concentrations for 48 h. The substrate MTT (Roche Diagnostics Corporation, Indianapolis, IN) was added to each well (e.g., add 10 μl of ready-use MTT substrate to 100 μl of cell culture medium) for 4 h according to the manufacturer’s protocol. Then 100 μl of the lysis buffer (provided in the kit) was added to each well containing MTT. After incubation at 37 °C overnight, the color development was measured by absorbance at 550 nm, with a reference absorbance at 690 nm. Each sample was assayed at least in triplicate. For the WST-1 assay, the cell seeding and treatment were the same as described in MTT assay. The substrate WST-1 (Cayman Chemical Company, Ann Arbor, MI) was added to each well (e.g., add 10 μl of ready-use WST-1 substrate to 100 μl of cell culture medium) for 2 h according to the manufacturer’s protocol. The color development was measured by absorbance at 450 nm. Each sample was assayed at least in triplicate. For the BrdU ELISA assay, the cell seeding and treatment were the same as described in MTT assay. 10 μM BrdU (Roche Diagnostics Corporation, Indianapolis, IN) was added during the last 4 h of the culturing period. After the cell culture medium was removed, cells were fixed for 30 min followed by incubation with anti-BrdU antibody for 1–2 h at 25 °C. After wash, the substrate was added and the color development was allowed for 15–30 min before measurement of absorbance at 450 nm, using absorbance at 670 nm as a reference. Each sample was assayed at least in triplicate.

### Cell Death Detection ELISA Assay

Cell lysates equivalent to 10^4^ cells after 48-hour treatment with or without HA (0–100 μg/ml) or HC-HA/PTX3 (0–100 μg/ml) were collected separately for assay (Cell Death Detection ELISA^PLUS^ Assay; Roche Applied Science) according to the manufacturer’s instructions. This ELISA is a photometric enzyme immunoassay for *in vitro* qualitative and quantitative determination of cytoplasmic histone-associated DNA fragments (mononucleosomes and oligonucleosomes) generated by apoptotic cell death using mouse monoclonal anti-histone and anti- DNA antibodies. The positive control was included as provided by the manufacturer, and absorbance was measured at 405 nm.

### Cell Migration

The migration assay was performed in 24-well transwell plate (8 μm pore size, Costar, Kennebunk, ME) by adding 0.5 ml DMEM/F12 (1:1) with or without EGF (10 ng/ml), FGF-2 (20 ng/ml), and TGF-β1 (10 ng/ml) in the lower compartment while adding 0.1 ml of ARPE-19 cells in DMEM/F12 (2 × 10^6^/ml) treated with PBS (vehicle control), HA (25 μg/ml), or HC-HA/PTX3 (25 μg/ml) to the upper compartment. After incubation at 37 °C for 8 h, cells not migrating through the pores were removed by a cotton swab, while cells on the filter facing the lower compartment were fixed with 5% glutaraldehyde, stained with 1% crystal violet, and counted from six random microscopic fields for each control or treatment group.

### Collagen Gel Contraction

0.25 ml of collagen type I solution (Corning, Bedford, MA) in cold DMEM/F12 (2.5 mg/ml) was added to each well of 24-well plates, followed by incubation at 37 °C for 1 h before adding 0.5 ml of ARPE-19 cells or primary human RPE cells (each at 5 × 10^5^/ml) with or without TGF-β1 (10 ng/ml) and PBS (vehicle control), HA (25 μg/ml), or HC-HA/PTX3 (25 μg/ml) on the top of collagen gel. After 24 h, the gels were freed from the walls of the culture wells with a small spatula. The photographic images of collagen gels were digitalized and the area was measured with NIH ImageJ 1.45 software. The percentage of gel contraction was determined by measuring the gel size at 72 h and comparing to the initial size (at 0 h).

### Extraction of Total RNA and Real-time PCR

Total RNAs were extracted using RNeasy Mini Kit (Qiagen, Valencia, CA) and reverse-transcribed using High Capacity Reverse Transcription Kit (Applied Biosystems, Foster City, CA). cDNA of each sample was amplified by quantitative real-time PCR using specific primer-probe mixtures (Col I α1: Hs00164004_m1; E-Cadherin: Hs01023894_m1; GAPDH: Hs02758991_g1; LEF1: Hs01547250_m1; ZEB1: Hs00232783_m1; ZEB2: Hs00207691_m1; α-SMA: Hs00426835_g1) and DNA polymerase in 7300 Real-time PCR System (Applied Biosystems, Foster City, CA). The program for real-time PCR profile consisted of 10 min of initial activation at 95 °C followed by 40 cycles of 15 sec denaturation at 95 °C, and 1 min of annealing and extension at 60 °C. The relative gene expression data were analyzed by the comparative CT method (ΔΔCT). All assays were performed in triplicates; the results were normalized by glceraldehyde-3-phosphate dehydrogenase (GAPDH) as an internal control.

### Immunofluorescence Confocal Microscopy

Cells were fixed in 4% paraformaldehyde/PBS (pH 7.0) for 15 min at room temperature. Then, cells were rehydrated in PBS, permeabilized in cold (−20 °C) acetone for 10 min at −20 °C. After being rinsed three times with PBS for 5 min each, cells were incubated with 5% BSA/0.03% Triton X-100/PBS for 30 min at room temperature to block non-specific binding. Appropriate primary antibodies were then added in 1% BSA/0.03% Triton X-100/PBS and incubated overnight at 4 °C. After three washes with PBS, cells were incubated with corresponding Alexa Fluor-conjugated secondary IgG for 60 min at room temperature. For BrdU staining, cells were fixed with 75% methanol plus 25% acetic acid for 15 min, denatured by 2 M HCl for 30 min at 37 °C and neutralized by washing in 0.1 M borate buffer, pH 8.5, three times for 5 min each. Monoclonal anti-BrdU and Alexa Fluor-conjugated secondary IgGs were used as described above. The sample incubated with an appropriate secondary antibody but not the primary antibody was used as a negative control. The samples were counterstained with Hoechst 33342 and analyzed with Zeiss LSM 700 confocal microscope (Thornhood, NY). Nuclear positive cells of β-catenin, LEF1, BrdU, or pSmad2/3 cytoplasm-positive α-SMA cells, and total cells, were counted from 4 random microscopic fields. The average percent (%) of the number of positive cells divided by the number of total cells in each field served as an index for comparison.

### Statistical Analysis

Quantitative results were expressed as mean ± S.D. from at least three independent experiments. Statistical significance for quantitative results was assessed using one-way analysis of variance followed by Tukey’s HSD post hoc test. In some cases, the two-tailed Student’s t test was used to assess significance. Differences were considered statistically significant when p < 0.05. Linear regression for comparison of WST-1 assay and BrdU ELISA data was generated in Excel 2013 (Microsoft Corp., Seattle, WA).

## Additional Information

**How to cite this article**: He, H. *et al*. Inhibition of Proliferation and Epithelial Mesenchymal Transition in Retinal Pigment Epithelial Cells by Heavy Chain-Hyaluronan/Pentraxin 3. *Sci. Rep.* **7**, 43736; doi: 10.1038/srep43736 (2017).

**Publisher's note:** Springer Nature remains neutral with regard to jurisdictional claims in published maps and institutional affiliations.

## Figures and Tables

**Table 1:** The commercial sources and doses of 18 growth factors and cytokines, designated as the “core factors” as reported by Pennock *et al*.^36^.

Growth factor	Type	Vendor	Catalog number	Effective *In vitro* dose	Dose used in this study
VEGF	human	R & D Systems	293-VE	2–50 ng/ml	50 ng/ml
PDGF-A	human	R & D Systems	221-AA	2–50 ng/ml	12.5 ng/ml
PDGF-AB	human	R & D Systems	222-AB	2–50 ng/ml	50 ng/ml
PDGF-B	human	R & D Systems	220-GMP	2–50 ng/ml	12.5 ng/ml
PDGF-C	human	R & D Systems	1687-CC	2–50 ng/ml	6.3 ng/ml
CTGF	human	Invitrogen	PHG0286	2–50 ng/ml	10 ng/ml
EGF	human	R & D Systems	236-EG	5–50 ng/ml	10 ng/ml
FGF-2	human	R & D Systems	233-FB	2–50 ng/ml	20 ng/ml
G-CSF	human	PeproTech	AF-300-23	5–75 ng/ml	50 ng/ml
HGF	human	R & D Systems	294-HGN	2–50 ng/ml	20 ng/ml
IFN-γ	human	PeproTech	AF-300-02	0.5–25 ng/ml	5 ng/ml
IGF1	human	R & D Systems	291-G1	2–50 ng/ml	10 ng/ml
IL-6	human	R & D Systems	200-06	0.2–10 ng/ml	10 ng/ml
MCP-1	human	PeproTech	AF-300-04	10–100 ng/ml	100 ng/ml
TGF-α	human	PeproTech	AF-100-16A	5–50 ng/ml	10 ng/ml
TGF-β1	human	PeproTech	AF-100-21C	2–20 ng/ml	10 ng/ml
TGF-β2	human	PeproTech	100-35	2–20 ng/ml	10 ng/ml
TGF-β3	human	PeproTech	AF-100-36E	2–20 ng/ml	10 ng/ml
